# Evaluation of cephalometric changes in Class II malocclusion following expansion vs. extraction orthodontic treatment: a comparative retrospective study

**DOI:** 10.25122/jml-2025-0157

**Published:** 2025-12

**Authors:** Nancy Ajwa, Othman AlOthman, Anas Baghareeb, Fatimah Radhi, Ibrahim AlMansour, Reham AlGhamdi, Hanan AlQahtani

**Affiliations:** 1 Preventive Dentistry Department, College of Medicine and Dentistry, Riyadh Elm University, Riyadh, KSA; 2 Private Practice, Riyadh, KSA; 3 Department of Periodontics & Orthodontics, School of Dental Medicine, University of Pennsylvania, Philadelphia, USA; 4 College of Medicine and Dentistry, Riyadh Elm University, Riyadh, KSA; 5 Majmaah University, Riyadh, KSA; 6 Ministry of Health, Al-Ahsa, KSA; 7 King Khalid University, Abha, KSA

**Keywords:** Class II malocclusion, cephalometric analysis, soft-tissue profile, orthodontic treatment, extraction therapy, expansion group

## Abstract

This study evaluated cephalometric changes in Class II malocclusion patients treated with expansion versus extraction in a Saudi Arabian sample. Data from 90 orthodontic patients meeting strict eligibility criteria were collected from multiple private practices in Saudi Arabia. The sample was divided according to treatment modality: Group 1 consisted of patients treated with four premolar extractions (*n* = 45), and Group 2 included patients treated with maxillary expansion using a banded rapid palatal expander (RPE) supported by mini-screws (*n* = 45). Nasolabial angle (NLA) for extraction cases presented a statistically significant difference in post-treatment radiographs (mean difference: -3.07 ± 8.92, *P* = 0.030) and significant changes in all dental variables (e.g., upper incisor position to A–Pog [UI-APog] pre-treatment mean difference: 4.49 ± 3.89; *P* < 0.001). In pre-treatment radiographs, only the position of the upper incisor to A-Pog showed a considerable difference (11.27 ± 4.19 vs. 8.83 ± 3.00, *P* = 0.017). Cases treated with RPE reported significant changes in the lower lip thickness (mean difference: -1.02 ± 2.20, *P* = 0.028). Cases treated with extraction had a greater influence on the dental component than on the soft tissue. In contrast, expansion cases showed a slight impact dentally but a greater effect on soft tissue parameters. However, neither treatment modality resulted in significant skeletal changes.

## INTRODUCTION

Facial esthetics, alongside occlusal correction, has become a primary focus of orthodontic treatment. Many patients now seek orthodontic care not only to improve function but also to enhance facial appearance, which can influence social interactions, self-esteem, and overall well-being [1,2]. Consequently, clinicians must be able to anticipate soft-tissue responses and treatment outcomes prior to intervention, particularly when addressing different types of malocclusions. Focusing on a Saudi sample is relevant due to ethnic variations in facial morphology, such as increased bimaxillary protrusion and convex profiles commonly observed in Middle Eastern populations, which may influence treatment responses differently than in Caucasian cohorts [3,4].

Class II malocclusion frequently presents with maxillary protrusion, with or without mandibular retrusion, and may be accompanied by vertical discrepancies. In addition, increased overjet and labially inclined incisors, due to either skeletal or dental manifestations, are also found [5,6].

There are several approaches to treating these discrepancies, such as maxillary expansion or extractions. Maxillary expansion is generally considered an early intervention strategy, as skeletal changes rely on modifying the growing maxilla. The mixed dentition phase is therefore ideal for rapid palatal expansion (RPE), which can address transverse deficiencies, correct posterior crossbites, and potentially eliminate the need for future extractions by increasing arch width [7,8]. RPE appliances—fixed or removable, rapid or slow—achieve expansion through activation of a midpalatal jackscrew. Mini-screw–supported expanders have gained popularity due to improved anchorage and skeletal effects. Following active expansion, the device must remain passively in place for at least three months to reduce relapse risk [9,10]. Furthermore, once the pubertal growth spurt has passed, the expansion approach is usually not applicable, and the extraction approach for permanent teeth is recommended.

Extraction is often required in cases of severe crowding or pronounced dental protrusion. Extraction patterns vary depending on the malocclusion: unilateral extraction is used for subdivision cases, whereas bilateral extraction is common in Class II patients [11]. First premolars are typically preferred for extraction because they provide adequate space relief; second premolars and molars are less commonly extracted because they offer limited space [12]. The extraction decision is guided by diagnostic criteria such as crowding severity, curve of Spee depth, incisor protrusion, vertical facial pattern, lip prominence, soft-tissue thickness, and anticipated effects on the facial profile [13].

Accordingly, lateral cephalometric radiograph assessment is an essential daily practice and a fundamental component of orthodontic procedures [2]. These systems enable orthodontists to anticipate soft-tissue and skeletal changes resulting from treatment and to better inform patients about their diagnosis and planned interventions [14-16]. In addition, advances in software technology have enabled cephalometric tracing with both digitizers and directly on screen-displayed digital images, enhancing accuracy and efficiency in orthognathic and orthodontic applications [14,16].

Numerous studies have discussed the impact of both treatment options for malocclusions on esthetics and occlusion. The debate regarding their effects on vertical dimensions, profile changes, jaw position, TMJ health, and periodontal status remains ongoing. Lip protrusion is an essential factor in determining the treatment decision. Previous studies have reported that the upper and lower lips tend to be more retruded in extraction cases compared with non-extraction groups, potentially impairing the facial profile [17,18]. Soft-tissue chin prominence has been less frequently reported than overall soft-tissue profile changes, while upper lip prominence appears to be the least affected [19,20]. Edward Angle was opposed to the extraction approach due to its complications with soft tissue changes and occlusal changes [4], a view challenged by recent reviews that highlight minimal long-term esthetic impacts [17]. Several lateral cephalometric measurements have been described and remain used to evaluate the harmony between skeletal, dental, and soft-tissue changes, and to compare pre- and post-treatment measurements [21-23].

Pointing out the controversy mentioned above, although there is extensive literature on soft-tissue changes following each treatment approach, there is minimal information on direct comparisons between them. Therefore, the purpose of this study was to digitally evaluate and compare cephalometric skeletal, dental, and soft-tissue changes using OnyxCeph software among Class II malocclusion patients treated with expansion vs. extraction modalities in a sample from the Kingdom of Saudi Arabia.

## MATERIAL AND METHODS

This retrospective study analyzed treatment records of 90 orthodontic patients, including 90 lateral cephalometric radiographs, with an average age of 16.60 years, representing different types of malocclusions and both genders. The sample size of 90 (45 per group) was retrospective; however, a post-hoc power analysis (G*Power 3.1) for key differences (e.g., UI-A-Pog, Cohen’s d = 0.67, α = 0.05) demonstrated a power greater than 0.90.

A consecutive sampling method was used, in which all patients who met the eligibility criteria during the data collection period were included. No selective inclusion or matching was performed, but both treatment groups were comparable in terms of age and gender distribution. Only patients with complete pre- and post-treatment cephalograms of diagnostic quality were included.

Eligibility selection criteria are shown in Table 1.

**Table 1:** Inclusion and exclusion criteria

Inclusion criteria	Exclusion criteria
Medically fit/non-syndromic subjects	History of trauma
Both genders	History of craniofacial problems
Bilateral extraction of all four premolars for orthodontic purposes.	History of growth modification
Treated cases using banded RPEs	Treatments, including TADs for anchorage
Class II skeletal malocclusion	Extractions for reasons other than orthodontics
Subjects aged 18 to 25 years	Surgical cases
Saudi patients only	Poor quality lateral cephalometric radiographs
Good-quality, clearly visible lateral cephalometric radiographs	Patients using medications that affect bone metabolism.
Patient not under any type of bone affecting medications	Non-Saudi patients
Availability of lateral cephalometric radiographs with adequate diagnostic quality	Congenitally missing teeth (excluding third molars)

Pre- and post-treatment digital lateral cephalometric radiographs were collected from multiple private practices in Saudi Arabia for each patient. The sample was divided into two main groups as follows:

**Group 1:** Four-premolar extraction cases treated with fixed appliances using reciprocal anchorage.

**Group 2:** Expansion cases treated with banded rapid palatal expanders (RPEs) supported by mini-screws.

Inter-investigator reliability was assessed using five lateral cephalometric tracings analyzed with Cohen’s Kappa test, yielding a score of K = 0.884, indicating adequate reliability. Intra-rater reliability was also evaluated (K = 0.912). The radiographs used in this pilot reliability assessment were not included in the final study sample. Magnification was eliminated by calibrating the ruler’s actual length against the head positioner using the software’s pixel-to-mm conversion.

Group 1 consisted of 45 patients (23 men, 22 women) treated with the extraction of four premolars in both the upper and lower jaws (*n* = 45) using reciprocal anchorage. Group 2 included 45 patients (23 men, 22 women) treated with banded RPEs supported by mini-screws.

**Figure 1:**
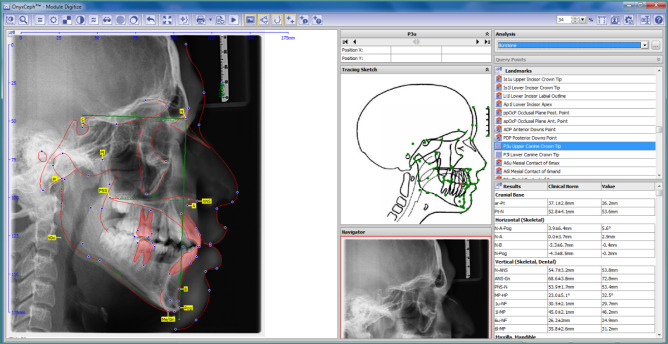
Skeletal and dental traced parameters

**Figure 2:**
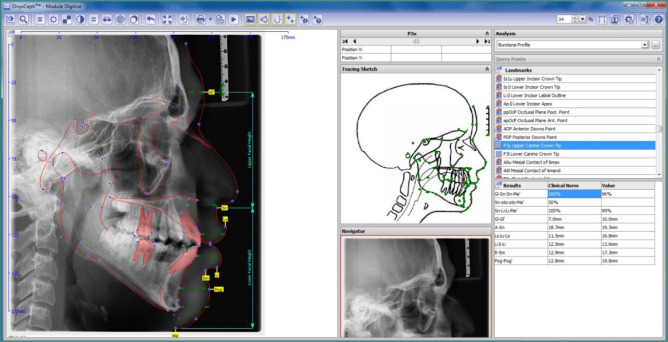
Soft tissue traced parameters

Lateral cephalograms were taken in centric occlusion with the lips at rest. Tracing was performed using OnyxCeph digital software (Image Instruments GmbH, Olbernhauer Str. 5, D-09125 Chemnitz, Germany) (M., 2009). Accuracy assessment was conducted to evaluate the risk of bias by one independent reviewer (NA). Specific skeletal, dental, and soft-tissue parameters were identified for cephalometric analysis. A total of 30 anatomical landmarks were marked, with five linear and ten angular measurements evaluated. These included four skeletal parameters and five dental parameters, as shown in Figure 1, and six soft-tissue parameters, as shown in Figure 2.

Comparison between selected soft-tissue measurements (following Burstone’s six linear measurements) [21], dental variables, and skeletal (hard-tissue) variables was performed. All skeletal, dental, and soft-tissue cephalometric variables are described in Table 2. Patient confidentiality was maintained by excluding names and personal identifiers; only gender and age were recorded for statistical analysis.

**Table 2:** Description of skeletal, dental, and soft-tissue cephalometric variables

**Skeletal variables**	FMA	The angle formed by the mandibular plane and the Frankfort plane.
	SNA	The angle formed by the SN plane and point A.
	SNB	The angle formed by the SN plane and point B.
	ANB	The angle formed by NA and NB.
**Dental variables**	UI- P	Angle formed by the intersection of the long axis of the upper incisor and the palatal plane.
	LI- M	Angle formed by the intersection of the long axis of the lower incisor and the mandibular plane.
	UI-LI	Angle formed between the planes of the upper and lower incisors.
	UI-APog	Perpendicular distance from the upper incisor tip to a line connecting A and Pogonion.
	LI-Apog	Perpendicular distance from the lower incisor tip to a line connecting A and Pogonion.
**Soft tissue variables**	NLA	Nasolabial angle formed by lines tangent to the upper lip and columella.
	G-G1	Linear distance between the G point (the most prominent point on the frontal bone) and the soft tissue, or analog point.
	A- SN	Distance between point A (the most concave point of the anterior maxilla) and the subnasale.
	Ls1u-Ls (J-LS)	Distance between the J point (the most labial point of the upper incisor) and the surface of the upper lip (labrale superius).
	Li1I-Li (I-LI)	Distance between the I point (the most labial point of the lower incisor) and the lower lip surface (labrale inferius).
	B-SM (B-ILS)	Distance between the B point (the most concave point on the mandibular symphysis) and the labiomental sulcus.
	Pog-Pog (PG-PG1)	Distance between the Pg point, the pogonion or the most prominent point of the chin, and the soft tissue-analog point.

FMA, Frankfort–mandibular plane angle; SNA, Sella–Nasion–A point angle; SNB, Sella–Nasion–B point angle; ANB, A–Nasion–B point angle; UI-P, Upper incisor to palatal plane angle; LI-M, Lower incisor to mandibular plane angle; UI-LI, Inter-incisal angle; UI-APog, Upper incisor to A–Pogonion distance; LI-APog, Lower incisor to A–Pogonion distance; NLA, Nasolabial angle; G–G1, Soft-tissue glabella thickness; A–SN, Point A to subnasale soft-tissue distance; Ls1u–Ls (J–LS), Upper lip thickness at upper incisor level; Li1I–Li (I–LI), Lower lip thickness at lower incisor level; B–SM (B–ILS), B-point to labiomental sulcus distance; Pog–Pog1 (PG–PG1), Pogonion to soft-tissue pogonion distance.

### Statistical analysis

Descriptive statistics, including frequency distributions and percentages, were calculated for categorical variables. Means and standard deviations were computed for all continuous skeletal, dental, and soft-tissue variables. Normality was confirmed using the Shapiro–Wilk test, where *P* > 0.05 indicated normally distributed data.

A paired *t*-test was used to compare mean skeletal, dental, and soft-tissue changes before and after treatment within the extraction and expansion groups. An independent *t*-test was used to compare the means between the two treatment groups. All analyses were performed using IBM SPSS (Version 25, Armonk, NY, USA), with statistical significance set at *P* < 0.05.

## RESULTS

A total of 90 cephalometric radiographs of orthodontic patients [women = 44 (48.8%) and men = 46 (51.1%)] treated either with palatal expansion [(*n* = 45), women = 22 (48.8%), men = 23 (51.1%)] or premolar extractions [(*n* = 45), women = 22 (48.8%), men = 23 (51.1%)] were analyzed to assess skeletal, dental, and soft-tissue changes. The mean age of the sample was 16.60 ± 4.59 years (Table 3).

Comparison of pre-treatment skeletal and soft-tissue variables did not show any significant differences between extraction and expansion cases. However, one dental variable demonstrated a significant difference prior to treatment: upper incisor position relative to A–Pog (UI-APog) was significantly higher in the extraction group (11.27 ± 4.19 mm) than in the expansion group (8.83 ± 3.00 mm; *P* = 0.017; Table 4).

Comparison of post-treatment skeletal and dental variables did not show any significant differences between extraction and expansion cases. However, a soft-tissue variable, nasolabial angle (NLA), showed a significantly higher value in extraction cases (104.52 ± 12.44) than in expansion cases 103.24 ± 13.26, *P* = 0.017 (Table 5).

The cephalometric analysis of pre-treatment versus post-treatment skeletal variables—Frankfort–mandibular plane angle (FMA), Sella–Nasion–A point angle (SNA), Sella–Nasion–B point angle (SNB), and A–Nasion–B point angle (ANB) did not show any significant mean differences (*P* > 0.05) in orthodontic cases treated with extractions. Dental variables showed clear, statistically significant differences: upper incisor inclination (UI-P) decreased from 121.25 ± 6.76° to 113.53 ± 8.32° (*P* < 0.001), and lower incisor inclination (LI-M) decreased from 6.20 ± 9.12° to 0.76 ± 8.77° (*P* = 0.001). Likewise, the upper incisor position to A-Pog (UI-APog) reduced from 11.27 ± 4.19 mm to 6.77 ± 2.63 mm (*P* < 0.001), while the lower incisor position relative to A-Pogonion (LI-APog) declined from 7.21 ± 4.79 mm to 3.60 ± 3.04 mm (*P* < 0.001). The inter-incisal angle (UI-LI) increased significantly (115.61 ± 12.34° to 128.65 ± 11.18°, *P* < 0.001) and showed a gender-related difference (*P* = 0.040). Soft-tissue changes were minimal, with only the B-point to labiomental sulcus distance (B-SM) showing a modest but significant reduction (13.52 ± 5.03 mm to 11.71 ± 2.07 mm; *P* = 0.025) (Table 6).

**Table 3:** Patient characteristics

Variables		*n*	%
Gender	Women	44	48.8%
	Men	46	51.1%
	Total	90	100.0%
Expansion vs. Extraction	Extraction	45	50%
	Expansion	45	50%
	Total	90	100.0%
Age (Mean ± SD)	16.60 ± 4.59 years		

**Table 4:** Comparison of pre-treatment variables between the extraction and expansion groups

Variables		Extraction		Expansion		*t*	P*
		Mean	SD	Mean	SD		
Skeletal	FMA	28.78	7.31	29.48	5.27	-0.41	0.686
	SNA	82.18	3.95	81.14	3.22	1.07	0.290
	SNB	79.12	3.17	78.18	3.27	1.11	0.273
	ANB	3.14	3.13	3.71	3.92	-0.61	0.544
Dental	UI- P	121.25	6.76	117.94	6.93	1.81	0.076
	LI- M	6.2	9.12	13.99	31.9	-1.18	0.247
	UI-LI	115.61	12.34	121.2	11.62	-1.74	0.087
	UI-APog	11.27	4.19	8.83	3	2.45	0.017
	LI-APog	7.18	4.64	5.29	3.5	1.69	0.096
Soft tissue	NLA	101.44	10.66	102.36	11.76	-0.31	0.758
	G-GI (G-GI)	8.28	10.19	6.34	1.9	0.94	0.352
	Ls1u-Ls (j-ls)	11.33	3.21	11.9	3.77	-0.62	0.539
	Li1I-Li(I-Li)	14.02	4.48	12.47	2.66	1.53	0.133
	B-SM(B-ILS)	13.52	5.03	11.89	2.18	1.51	0.137
	Pog-Pog’ (PG-PG1)	13.15	4.36	12.34	2.57	0.82	0.414

*Independent *t*-test

No significant skeletal alterations were found (*P* > 0.05 for all skeletal parameters). Dental findings indicated mild yet significant changes: the inter-incisal angle (UI-LI) increased from 121.20 ± 11.62° to 126.91 ± 10.51° (*P* = 0.039), and the UI-APog decreased from 8.83 ± 3.00 mm to 6.66 ± 2.87 mm (*P* < 0.001), indicating minor uprighting and retrusion of the incisors. Soft-tissue evaluation revealed significant increases in both upper and lower lip thickness—Ls1u–Ls (upper incisor labial point to labrale superius) increased from 11.90 ± 3.77 mm to 13.62 ± 3.24 mm (*P* = 0.020), and Li1I–Li (lower incisor labial point to labrale inferius) increased from 12.47 ± 2.66 mm to 13.50 ± 2.41 mm (*P* = 0.028)—reflecting subtle lip fullness following expansion. All other soft-tissue parameters remained stable (Table 7).

## DISCUSSION

One of the main concerns in orthodontic treatment is esthetics. Therefore, choosing the treatment modality is crucial due to its effects on appearance. Many claims about the premolar extraction approach suggest a deleterious impact on the face, including a flattened facial profile due to retruded upper and lower lips [24]. However, this study compares the changes between pre- and post-treatment radiographs for different treatment modalities—premolar extraction versus expansion via banded hyrax—skeletally, dentally, and in the soft tissues.

To begin, skeletal changes were not evident in either the extraction or the expansion approaches. The position of the maxilla and mandible, and their relationship, remained nearly the same. These results are predictable given the absence of bone-modifying appliances in the extraction protocol and the bone maturation at this stage of life. Conversely, all dental variables showed significant differences between the extraction methods. Both upper and lower incisors protruded by 4.49 mm and 3.61 mm, respectively, and retroclined by 7.71° and 5.44°, respectively, and the angle between the incisors decreased by –13.03°. These data are in agreement with previous findings [25].

**Table 5:** Post-treatment comparison between the extraction and expansion groups

Variables		Extraction		Expansion		*t*	P*
		Mean	SD	Mean	SD		
Skeletal	FMA	28.98	7.29	28.94	6.83	-0.367	0.715
	SNA	80.13	11.01	81.24	3.63	1.451	0.152
	SNB	79.48	3.28	78.11	3.18	-0.110	0.913
	ANB	2.69	3.18	3.80	4.00	0.997	0.323
Dental	UI- P	113.53	8.32	110.88	22.94	0.473	0.638
	LI- M	0.76	8.77	3.08	7.95	-0.979	0.332
	UI-LI	128.65	11.18	126.91	10.51	1.806	0.076
	UI-APog	6.77	2.63	6.66	2.87	-1.853	0.069
	LI-APog	3.60	3.04	4.44	2.85	-1.960	0.055
Soft tissue	NLA	104.52	12.44	103.24	13.26	2.222	0.030
	GI-GI’ (G-GI)	5.90	1.47	6.33	1.82	0.592	0.557
	Ls1u-Ls (j-ls)	11.09	2.71	13.62	3.24	-0.628	0.532
	Li1I-Li(I-Li)	12.97	2.34	13.50	2.41	-0.134	0.894
	B-SM(B-ILS)	11.71	2.07	12.16	2.68	-0.519	0.606
	Pog-Pog’ (PG-PG1)	11.57	3.33	12.88	2.88	0.064	0.949

*Independent *t*-test

**Table 6:** Pre-treatment and post-treatment comparison of cephalometric variables in extraction cases

Variables		Extraction						*P* ^¶^
		Pre-treatment		Posttreatment		Mean Diff		
		Mean	SD	Mean	SD	Mean	SD	
Skeletal	FMA	28.78	7.31	28.98	7.29	-0.21	3.12	0.711
	SNA	82.18	3.95	80.13	11.01	2.04	11.11	0.306
	SNB	79.13	3.17	79.48	3.28	-0.36	2.35	0.393
	ANB	3.14	3.13	2.69	3.18	0.45	2.26	0.272
Dental	UI- P	121.25	6.76	113.53	8.32	7.71	10.21	<0.001
	LI- M	6.20	9.12	0.76	8.77	5.44	8.38	0.001
	UI-LI^§^	115.61	12.34	128.65	11.18	-13.03	14.03	<0.001
	UI-APog	11.27	4.19	6.77	2.63	4.49	3.89	<0.001
	LI-APog	7.21	4.79	3.60	3.04	3.61	4.72	<0.001
Soft tissue	NLA	101.44	10.66	104.52	12.44	-3.07	8.92	0.061
	GI-GI’ (G-GI)	8.28	10.19	5.90	1.47	2.38	10.27	0.199
	Ls1u-Ls (j-ls)	11.33	3.21	11.09	2.71	0.24	3.85	0.729
	Li1I-Li(I-Li)	14.02	4.48	12.97	2.34	1.05	4.72	0.218
	B-SM(B-ILS)	13.52	5.03	11.71	2.07	1.81	4.34	0.025
	Pog-Pog (PG-PG1)	13.15	4.36	11.57	3.33	1.58	4.67	0.065

¶ paired *t*-test. §in extraction cases the mean difference was significant [(women = -18.06 ± 12.88) vs (men = -8.00 ± 13.66), *P* = 0.040)]

**Table 7:** Pre-treatment and post-treatment comparison of cephalometric variables in expansion cases

Variables		Expansion						*P* ^¶^
		Pre-treatment		Posttreatment		Mean Diff		
		Mean	SD	Mean	SD	Mean	SD	
Skeletal	FMA	29.48	5.27	28.94	6.83	0.54	4.28	0.534
	SNA	81.14	3.22	81.24	3.63	-0.11	2.30	0.816
	SNB	78.18	3.27	78.11	3.18	0.06	1.42	0.824
	ANB	3.71	3.92	3.80	4.00	-0.10	1.47	0.748
Dental	UI- P	117.94	6.93	110.88	22.94	7.07	24.43	0.161
	LI- M	13.99	31.90	3.08	7.95	10.91	30.99	0.091
	UI-LI	121.20	11.62	126.91	10.51	-5.70	13.08	0.039
	UI-APog	8.83	3.00	6.66	2.87	2.17	2.66	<0.001
	LI-APog	5.29	3.50	4.44	2.85	0.85	2.45	0.095
Soft tissue	NLA	102.36	11.76	103.24	13.26	-0.87	9.69	0.657
	GI-GI’ (G-GI)	6.34	1.90	6.33	1.82	0.01	0.68	0.930
	Ls1u-Ls (j-ls)	11.90	3.77	13.62	3.24	-1.72	3.44	0.020
	Li1I-Li(I-Li)	12.47	2.66	13.50	2.41	-1.02	2.20	0.028
	B-SM(B-ILS)	11.89	2.18	12.16	2.68	-0.27	2.31	0.568
	Pog-Pog’ (PG-PG1)	12.34	2.57	12.88	2.88	-0.54	2.33	0.255

¶ paired *t*-test

In contrast, in the expansion modality, the analyzed data showed a slight increase in the angle formed between the upper and lower incisor planes (UI-LI); in addition, a significant retrusion of the upper incisors was noted. Soft-tissue changes were generally not substantial, except for changes in the labiomental sulcus depth. These findings are consistent with previous studies that did not report significant differences in the NLA. However, those studies did observe an increase in NLA with both 2- and 4-premolar extraction protocols, as both lips tend to retract, with less retraction of the lower lip in the 2-premolar extraction protocol [14,11].

Moreover, labiomental sulcus depth was reduced in the post-treatment radiographs. The interincisal gap and facial convexity were measured and compared, as their inclusion criteria included only Class I bimaxillary protrusion cases [25]. They noted a positive correlation between changes in the NLA and the labiomental sulcus depth. Similarly, Janson reported an increase in NLA in response to lip retraction, although his sample included only Class II cases; therefore, the effect on the anteroposterior component was more pronounced [17]. These data refute the hypothesis that premolar extraction necessarily harms facial esthetics.

Nevertheless, increased upper and lower lip thicknesses were observed in the expansion group, by 1.72 mm and 1.02 mm, respectively. These results contrast with the findings of Kim *et al.* [26], who reported a decrease in both lip thicknesses following expansion. The observed difference in our study may be attributed to the use of mini-screw anchorage, which potentially reduces transverse soft-tissue stretching, or to population-specific soft-tissue adaptations within the Saudi sample. Kim *et al.* attributed their results to transverse expansion and stretching of perioral soft tissues, and they relied on CBCT for assessment, which provides greater measurement accuracy than conventional cephalometry [26]. No other significant differences were noted in the present analysis.

Although several variables demonstrated statistically significant differences, the magnitudes of change, particularly for soft-tissue parameters such as lip thickness (approximately 1-2 mm), may not be clinically perceptible. These findings indicate that, while orthodontic treatment can cause measurable dimensional variations, its esthetic impact is likely subtle. Therefore, statistical significance should be interpreted in light of clinical relevance and patient perception when planning treatment.

However, there were no significant differences in the pre-treatment radiographs between the two groups, except for the upper incisor position (UI-APog). The incisors were more retruded in the extraction group, and the post-treatment results showed significant differences only in the NLA, with a higher angle observed in the extraction group. Clinically, extraction may be more suitable for cases with severe protrusion requiring dental retrusion, whereas expansion tends to preserve soft-tissue characteristics. Future studies should consider using CBCT to allow for more comprehensive three-dimensional evaluation.

### Limitations

Limitations include a retrospective design (potential selection bias), limited long-term relapse assessment, and unaccounted confounders (e.g., treatment duration, operator variability).

## CONCLUSION

Within the limitations of the current study, extraction influenced dental components more than soft tissue, while expansion had slight dental but greater soft tissue effects. No skeletal changes were noted in either modality. Future research should explore long-term outcomes with 3D imaging.

## Data Availability

The datasets generated and analyzed during this study are available from the corresponding author upon reasonable request.
